# Lemierre's Syndrome: Rare, but Life Threatening—A Case Report with *Streptococcus intermedius*

**DOI:** 10.1155/2012/624065

**Published:** 2012-11-04

**Authors:** Shalini Gupta, Shehzad S. Merchant

**Affiliations:** ^1^Department of Internal Medicine, Oregon Health Science University, P.O. Box BTE 119, 3181 SW Sam Jackson Park Road, Portland, OR 97239, USA; ^2^Infectious Diseases Division, Department of Medicine, Buffalo General Hospital, University at Buffalo, 100 High Street, B-8, Buffalo, NY 14203, USA

## Abstract

Lemierre's syndrome (LS) is a rare, but a life-threatening complication of an oropharyngeal infection. Combinations of fever, pharyngitis, dysphagia, odynophagia, or oropharyngeal swelling are common presenting symptoms. Infection of the lateral pharyngeal space may result in thrombosis of the internal jugular vein, subsequent metastatic complications (e.g., lung abscesses, septic arthritis), and significant morbidity and mortality. LS is usually caused by the gram-negative anaerobic bacillus *Fusobacterium necrophorum*, hence also known as necrobacillosis. We present a case of LS caused by *Streptococcus intermedius*, likely secondary to gingival scraping, in which the presenting complaint was neck pain. The oropharyngeal examination was normal and an initial CT of the neck was done without contrast, which likely resulted in a diagnostic delay. This syndrome can be easily missed in early phases. However, given the potential severity of LS, early recognition and expedient appropriate antimicrobial treatment are critical. *S. intermedius* is an unusual cause of LS, with only 2 previous cases being reported in the literature. Therefore, an awareness of the myriad presentations of this syndrome, which in turn will lead to appropriate and timely diagnostic studies, will result in improved outcome for LS.

## 1. Introduction 

Lemierre's syndrome (LS) is a life-threatening, but a rare complication of an oropharyngeal infection [1]. In the preantibiotic era, Lemierre's syndrome was associated with a case-mortality rate of 32%–90%, with embolic events in 25% and endocarditis in 12.5% of the patients. It is still a potentially life-threatening disease with a reported mortality of up to 17% [2].

In the post antibiotic era, it was named the “forgotten disease” until recently, when it started presenting more frequently and uniquely. The suggested diagnostic criteria are (1) history of recent oropharyngeal infection, (2) clinical or radiographic evidence of thrombophlebitis of the internal jugular vein (IJV), and (3) isolation of an anaerobic pathogen [1, 3]. LS usually presents as a sore throat, and pharyngitis is the entry source for more than 85% of cases, while otitis media or dental infection accounts for <2% of cases [1, 4]. As the disease progresses, the soft tissues of the neck are invaded by anaerobic oral pathogens, followed by local invasion of the lateral pharyngeal space and septic thrombophlebitis of the internal jugular vein (IJV). It may lead to septic emboli and metastatic abscess, especially in the lungs and joints. Complications like meningitis, osteomyelitis, splenic abscesses, cranial nerve involvement, carotid thrombosis, and mediastinitis have been reported [3, 5, 6]. 

LS is usually caused by the gram-negative anaerobic bacillus *Fusobacterium necrophorum, *hence also known as necrobacillosis [1, 4]. Other etiological agents like *Peptostreptococcus*, Group B and C *Streptococcus*, *Staphylococcus*, *Enterococcus* species and Proteus have also been isolated [1, 7, 8]. Fusobacterium is a natural colonizer in the oropharynx of healthy adults. However, pharyngitis weakens the mucosal barrier and allows Fusobacterium to enter the bloodstream and cause complications. Early diagnosis with imaging and blood cultures in clinically suspicious patients can prevent mortality and morbidity.

*S*. *intermedius*, one of the member of *Streptococcus* milleri group, is a microaerophilic commensal found commonly in the upper respiratory and gastrointestinal tract and is capable of causing pyogenic infections especially in the liver, brain and skin [8, 9] and most importantly the heart valves [10]. To the best of our knowledge, this is the first case report of LS with *Streptococcus intermedius* in an immunocompetent adult resulting from a gingival procedure with a normal oropharyngeal examination at the time of presentation. 

## 2. Case Presentation

A middle-aged woman presented to the emergency room (ER) with complaints of severe neck pain and occipital headaches for one week, which were not relieved with analgesics. She denied fevers, sore throat, cough, shortness of breath, or any trauma to the neck. Past medical history was significant for epilepsy and prior episodes of supraventricular tachycardia (SVT). She denied smoking or illicit drug use. She had a dental scraping of her left mandibular molars two weeks prior to gingivitis. Vital signs were stable in ER. Physical exam was positive for neck tenderness and minimal restriction of neck movements. Laboratory data revealed a white blood cell (WBC) of 11.9 × 10^3^/mm^3^ (neutrophils 79%). Computerized tomography (CT) scan of the head and neck without contrast and lumbar puncture were done to rule out subarachnoid hemorrhage. The results did not reveal any abnormalities. Hence the patient was discharged on muscle relaxants and analgesics.

She returned to the ER in 5 days with high grade fevers, worsening neck pain and a headache. Temperature was 101°F, heart rate 162/min, respiratory rate 16/min, blood pressure 152/96 mmHg, and oxygen saturation of 92% on room air. Pharyngeal exam showed no erythema, swelling, or exudates. There was no evidence of otitis media or an active gingivitis either. There was no dental caries noted at that time, and there was no heat or cold intolerance. Percussion tenderness was not present. Neck examination showed restriction in range of movements, and a tender cord-like mass was palpable on the left side of neck. Cardiopulmonary examination revealed diffuse crackles in both lungs and no cardiac murmurs. 

**Figure 1:**
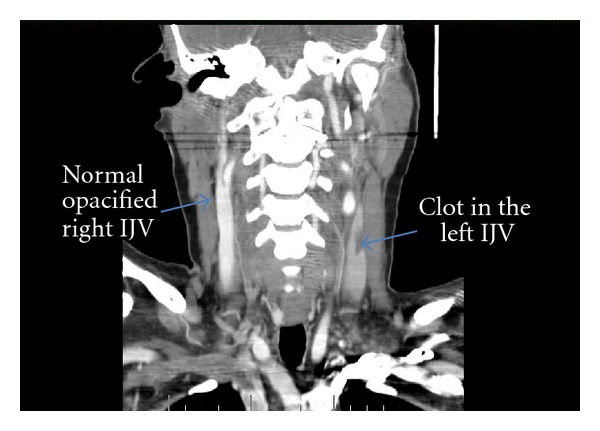
CT scan of the head and neck with contrast demonstrated a thrombus in the left internal jugular vein (IJV).

**Figure 2:**
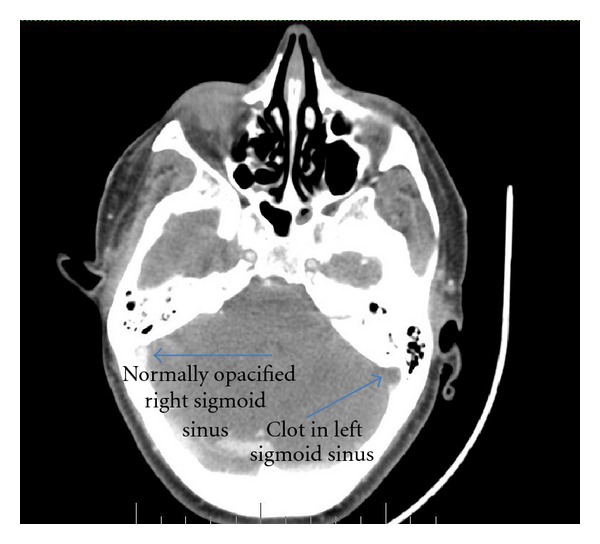
CT scan of the head and neck with contrast demonstrated a thrombus in the left sigmoid sinus.

**Figure 3:**
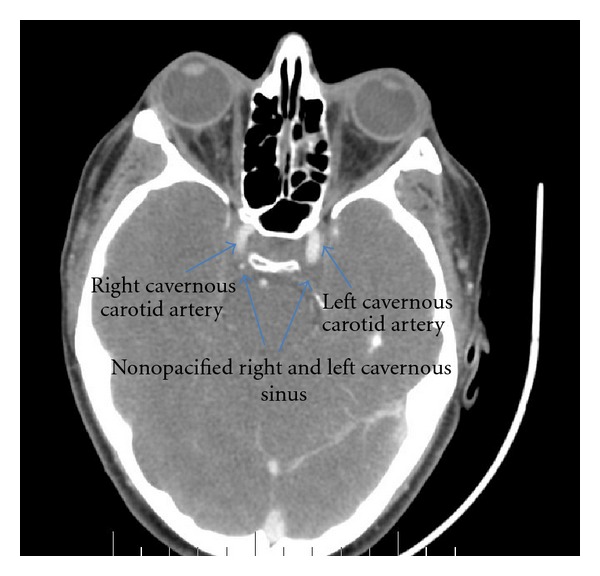
CT scan of the head and neck with contrast demonstrated a thrombus bilaterally into the cavernous sinuses.

Laboratory data showed a WBC count of 33.6 × 10^3^/mm^3^ (bands 44%) and an ESR of 98 mm/hr. Complete metabolic profile (CMP), including electrolytes, renal function (BUN, creatinine), and liver enzymes (LFTs) were all within normal range. EKG showed SVT. Chest X-ray showed small bilateral pleural effusions and bilateral pulmonary infiltrates without cavitations. CT scan of the head and neck with contrast demonstrated a thrombus in the left internal jugular vein (IJV) (Figure 1) extending to left sigmoid sinus (Figure 2) and bilaterally into the cavernous sinus (Figure 3). There was diffuse edema around the soft tissues of the neck. Preliminary blood cultures grew gram-positive cocci in chains. She was started empirically on intravenous Clindamycin and Vancomycin. Unfractionated heparin was also started due to the extensive clot burden. Workup for autoimmune and hypercoagulable diseases was unrevealing for any abnormality. Autoimmune workup included anti-nuclear anti-bodies (ANA), rheumatoid factor (RF), cytoplasmic antineutrophil cytoplasmic antibodies (c-ANCA), perinuclear anti-neutrophil cytoplasmic antibodies (p-ANCA), anti-Ro/SSA and anti-La/SSB antibodies. Hypercoagulable workup included prothrombin time (PT), partial thromboplastin time (PTT), international normalized ratio (INR), factor V Leiden mutation, prothrombin gene mutation, protein C and S as well as antithrombin levels, anti-cardiolipin antibodies and lupus anticoagulant.

Blood cultures (4 out of 4) grew *Streptococcus intermedius *within 48 hours, and the organism was found to be highly susceptible to penicillin (but also susceptible to clindamycin and vancomycin). Subsequently, antibiotics were changed to ampicillin-sulbactam. A blood culture six days after admission also grew the same organism. 2D echocardiogram did not reveal any valvular vegetations. Anticoagulation was stopped after 10 days due to a drop in hemoglobin (from 10.5 g/dL to 6.9 g/dL), although Esophagogastroduodenoscopy (EGD), colonoscopy, and a CT scan of the abdomen and pelvis did not reveal an obvious bleeding source. Serum LDH, reticulocyte and haptoglobin were within normal limits, hence arguing against hemolysis. Ampicillin-sulbactam was continued for eight weeks, and the patient had a slow but complete clinical recovery with radiographic resolution of the clot.

## 3. Discussion

In summary, we present a middle-aged female who presented with severe neck pain and occipital headaches for a week who did not have fevers or a sore throat on initial presentation but did provide a history of dental work performed two weeks ago. A noncontrasted CT of the head and neck failed to reveal any pathology on admission, and she was discharged from the ER whereby she very rapidly deteriorated over the next five days and then presented with worsening fevers, headaches, neck pain, and a leukocytosis of 34,000. Five blood cultures performed over a period of six days grew *Streptococcus intermedius*, and a CT head and neck with contrast showed thrombosis of the jugular vein, cavernous sinuses, and left sigmoid sinus. Patient survived and recovered after eight weeks of ampicillin-sulbactam. 

Lemierre's syndrome is a rare disease, typically caused by the microorganism *Fusobacterium necrophorum.* Tonsillitis is the most common primary infection (87.1%) followed by mastoiditis (2.7%) and odontogenic infections (1.8%) [11, 12]. This is typically followed by invasion of the pharyngeal lateral wall and thrombophlebitis of the internal jugular vein followed by high grade bacteremia and septic seeding of vital organs, most commonly the lungs. It is quite likely that our patient developed LS secondary to the gingival scraping that she underwent two weeks before her symptoms started.* S*. *intermedius* is a rare causative organism, and only 2 case reports of LS were found with this bacterium [8, 13]. Escalona et al. [8] reported a case with LS due to *S. intermedius*. This patient presented with extensive mandibular swelling due to an infected molar and fevers, along with an edematous floor of the mouth on physical exam. Chemlal et al. [13], reported a patient with LS, who had a recent pharyngitis presenting with fever and lower chest pain related to multiple pulmonary abscesses. Pharyngitis is a single most common presentation of LS as mentioned by Wright et al. [14]. In contrast, our patient's mouth examination was totally benign, and she did not have a history of sore throat in the recent past. Our patient's benign presentation and normal oropharyngeal examination might have delayed her diagnosis. It is tempting to speculate that differences in virulence properties between *S. intermedius* and* F. necrophorum* which is the usual pathogen responsible for LS may have contributed to an atypical presentation. However, the previous 2 reported cases of *S. intermedius* [8, 13] did present with oropharyngeal signs. Therefore, it is important to recognize that LS can present without any signs of pharyngitis or an active dental or ear infection and hence can be missed in the early phases of infection [3].

Diagnosis using CT scan of the head and neck with IV contrast is considered superior to a neck ultrasound as it is better in locating the anatomical extension of the thrombus [4, 5]. CT scan in the absence of contrast may be of limited utility (as was the case in our patient). Blood cultures should be sent on a patient with persistent severe pharyngitis and signs of sepsis and even in patients presenting with fevers and severe neck pain. Penicillin is the drug of choice, but due to recent penicillin-resistant strains of Fusobacterium, drugs like Clindamycin or beta lactam/beta-lactamase inhibitor are preferred [7, 14, 15]. Therapy should be started as soon as the syndrome is suspected and should be continued for at least 6 weeks [14–16]. Surgical drainage of abscess and IJV ligation may be indicated for patients who fail to respond to antibiotics, as was done in the preantibiotic era, though the ligature is not frequently done now [4, 8]. Routine use of anticoagulation is controversial as there are no randomized trials, and sepsis-related thrombocytopenia is often seen in these cases [14, 16]. Anticoagulation should strongly be considered, if there is clot propagation involving the cavernous sinus or if there are septic emboli [4, 5, 7]. However anticoagulation can increase the risk of bleeding and expansion of hematoma.

## 4. Summary

Lemierre's syndrome usually presents in childhood but may present atypically in middle-aged people, as in our patient. It can happen after pharyngitis, otitis media, odontogenic infections, or dental procedures [4]. The number of reported cases is increasing, due to the restricted use of antibiotics for sore throat and tonsillitis [5, 7]. High grade bacteremia with *Streptococcus intermedius *due to septic thrombosis, without any signs of an oral or pharyngeal infection at the time of presentation, is a unique feature of this case. Further, the ability of other oral flora to be causative agents of Lemierre's syndrome is not as well established and recognized as it is with *Fusobacterium necrophorum*. This suggests that a benign mouth exam should not exclude the diagnosis of LS. In light of this, we recommend that LS should be considered in the differential diagnosis in patients presenting with persistent sore throat, mastoiditis, recent history of a dental procedure, and/or signs of active gingivitis, accompanied with neck pain and swelling. Blood cultures should be obtained and CT imaging of the neck with IV contrast should be performed. This, in turn, will enable timely diagnosis and improved outcome.
